# From bench to bowel: translating targeted drug delivery through gastrointestinal barriers

**DOI:** 10.3389/fbioe.2026.1763085

**Published:** 2026-07-15

**Authors:** Zhou Zhang, Chenxin Wang, Jinhao Zhang, Fangqian Wang, Xiaohua Yu, Liangliang Yu

**Affiliations:** 1 Department of Endoscopy Center, Sir Run Run Shaw Hospital, Zhejiang University School of Medicine, Hangzhou, China; 2 Department of Orthopedic Surgery the Second Affiliated Hospital, Zhejiang University School of Medicine, Hangzhou, China; 3 Orthopedics Research Institute of Zhejiang University, Hangzhou, China; 4 Key Laboratory of Motor System Disease Research and Precision Therapy of Zhejiang Province, Hangzhou, China; 5 Clinical Research Center of Motor System Disease of Zhejiang Province, Hangzhou, China

**Keywords:** gastrointestinal barriers, gastrointestinal cancer, gastrointestinal diseases, IBD, nanomedicine, oral drug delivery, stimuli-responsive systems, targeted drug delivery

## Abstract

The complex and heterogeneous physiological barriers of the gastrointestinal (GI) tract severely limit the efficacy of therapeutic agents for local diseases. This review comprehensively examines strategies for translating targeted drug delivery “from bench to bowel” within the GI tract. We detail the unique biological barriers across GI segments (oral cavity, esophagus, stomach, small intestine, colon) and analyze core challenges in designing effective delivery systems: payload stability, precise targeting/retention, controlled release, mucosal/epithelial penetration, and biocompatibility. The article critically evaluates recent advancements in key delivery platforms, including nanocarriers (liposomes, polymeric NPs, dendrimers, inorganic NPs), hydrogels, and microscale systems (microspheres, microneedles, microrobots), categorizing them by their targeting mechanisms: passive (e.g., the classical EPR effect in solid tumors and EPR-like permeability/retention phenomena in inflamed GI lesions), passive (e.g., EPR effect), active (ligand-mediated), and stimuli-responsive (pH, enzymes, redox, etc.). Furthermore, we highlight the transformative applications of these targeted systems in treating major GI diseases such as inflammatory bowel disease (IBD), GI cancers, and peptic ulcer disease, emphasizing their potential to enhance local efficacy while minimizing systemic toxicity. This work bridges fundamental principles with translational progress to provide a roadmap for developing clinically viable targeted therapies for GI diseases.

## Graphical Abstract

**Figure:**
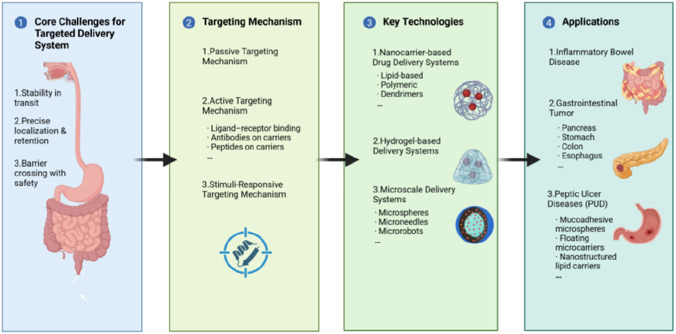
A conceptual graphical abstract is provided below.

## 1 Introduction

**FIGURE 1:**
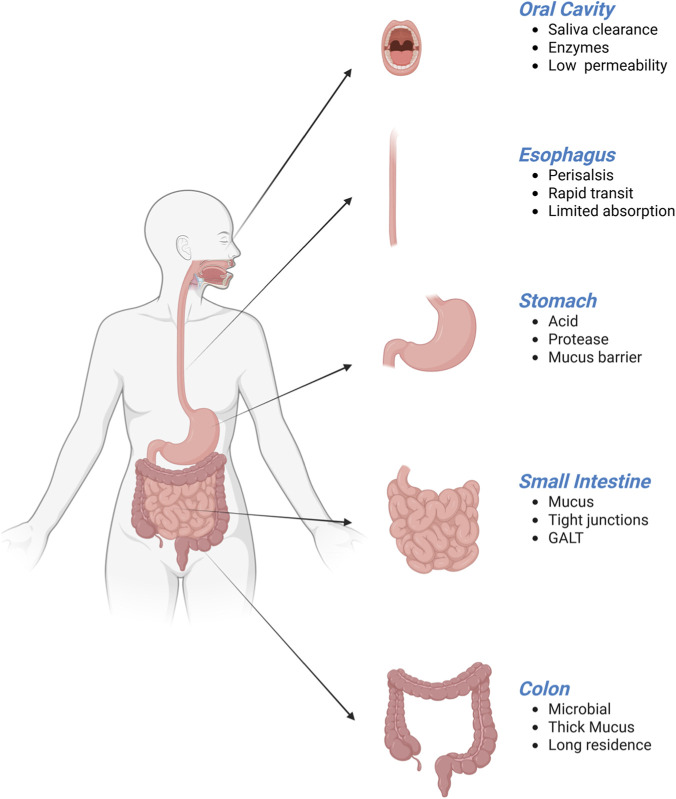
Region-specific physiological barriers in the gastrointestinal tract relevant to oral targeted drug delivery (Detailed regional parameters and definitions are summarized in Table 1).

Oral drug delivery remains the most patient-preferred administration route due to its non-invasiveness, convenience, and potential for high compliance (Drucker, 2020). However, the GI tract is a highly evolved defensive system for digestion and absorption, and its layered physiological and biochemical barriers severely limit the efficacy of therapeutic agents—especially biologics and therapies requiring site-specific deposition (Wang et al., 2023). The oral bioavailability of intact peptides and proteins is <1% and sometimes even <0.1% (Brown et al., 2020). Importantly, these barriers are region-dependent rather than uniform, meaning that “one-formulation-fits-all” strategies rarely translate across diseases and GI segments (Figure 1; Table 1). While the stomach imposes harsh acidity (pH 1.0–4.0) and proteolytic enzymes that destabilize acid-labile drugs and proteins, the small intestine—despite being the principal absorption site—adds coupled hurdles including dense mucus, potent enzymatic activity, tight epithelial junctions, efflux pumps, and immune surveillance (Lundquist and Artursson, 2016). Mucus protects the underlying epithelium by lubricating the surface and trapping/removing foreign particulates (Ensign et al., 2012), and goblet-cell-derived extracellular mucus can directly hinder nanoparticle transport and access to absorptive epithelium (Wang Y. et al., 2024). The colon, although offering near-neutral pH and long residence time, still presents a thick mucus barrier and a microbiota-rich environment capable of drug degradation. Moreover, once nanoparticles encounter GI fluids, biomolecule adsorption generates a “protein corona” that can reshape surface identity and thereby alter mucus interaction, epithelial uptake, and targeting outcomes (Zheng et al., 2024).

**TABLE 1:** Physiological characteristics and drug delivery barriers.

GI region	Physiological characteristics	Drug delivery barriers	Key challenges	Potential solutions
Oral Cavity	* Near-neutral pH (6.6–7.1) * Rich vascularization * Stratified squamous epithelium	* Extremely short transit time * Constant salivary clearance * Degradative enzymes * Low permeability of buccal mucosa	*Insufficient absorption time *Limited to small lipophilic molecules	*Mucoadhesive systems *Permeation enhancers *Enzyme inhibitors
Esophagus	* Primarily a conduit *via* peristaltic movements * Thin protective mucus layer * Stratified squamous epithelium	* Very limited surface area * Minimal blood supply * Short transit time * Mucus layer acts as a minor physical barrier	*No practical absorption *Risk of local irritation	*Rapid transit formulations *Protective coatings
Stomach	* Extreme acidity * Mechanical churning * Secretes gastric juice * Thick mucus-bicarbonate layer	* Acidic Degradation * Enzymatic Barrier * Mucus-Bicarbonate Barrier * Tight Junctions	*Destruction of acid-labile drugs and proteins *Limited absorption	*Enteric coatings *Enzyme inhibitors *Gastroretentive systems *pH-responsive polymers
Small Intestine	* Primary site for nutrient absorption * Vast surface area * Relatively prolonged transit time * Near-neutral pH * Contains Gut-Associated Lymphoid Tissue (GALT)	* Powerful Enzymatic Barrier * Dense Mucus Layer * Epithelial Barrier * Tight Junctions * Transcellular Limitations * Efflux Transporters * Immune Barrier	*Enzymatic degradation *Mucus entrapment *Poor epithelial permeability *Active efflux *Immune clearance	*Enzyme-resistant formulations *Mucus-penetrating particles (<200 nm, PEGylated) *Permeation enhancers *Efflux inhibitors *Immune-evasive coatings
Colon	* Near-neutral pH * Longest residence time * Highly diverse and dense microbiota * Thicker mucus layer than SI	* Microbiota and Enzymatic Challenge * Thick Mucus Barrier	*Microbial drug metabolism *Mucus barrier *Lower permeability	*pH-dependent polymers *Time-dependent systems *Microbially-triggered carriers *Mucoadhesive/mucus-penetrating formulations

Recent advances in nanotechnology, biomaterials science, and bioengineering have spurred the development of sophisticated targeted drug delivery systems (DDS) to navigate the complex GI environment. Yet, while drug delivery efficiency is severely hindered by complex intestinal barriers—particularly mucus and epithelium (Wang et al., 2025)—current research is often fragmented, with many studies emphasizing either materials innovation or disease application without explicitly connecting (i) the clinical disease context, (ii) the dominant physiological barrier(s), and (iii) the corresponding carrier design and targeting mechanism (Xiao et al., 2021). A clinically actionable framework should map barrier “pain points” to engineering “solutions”: for example, ulcerative lesions demand mucus/epithelial interaction control and localized retention; inflamed/tumor tissues may permit passive leveraging of altered permeability (Yao et al., 2024); and pH/enzymes/redox gradients can be harnessed for on-site activation (Zhu et al., 2024)—each requiring distinct nanocarrier choices and mechanistic targeting logic. Accordingly, targeted systems can be broadly categorized by carrier platform—nanocarriers (liposomes, polymeric nanoparticles, dendrimers, inorganic NPs), hydrogels, and microscale systems (microspheres, microneedles, microrobots)—and by targeting mechanism: passive leveraging (e.g., EPR-like accumulation in inflamed or tumor tissue), active (ligand/receptor-mediated uptake), or stimuli-responsive (endogenous triggers such as pH, enzymes, redox; or exogenous triggers such as light/magnetism) (Li et al., 2025). Clarifying “which barrier is being solved, in which disease, by which mechanism” is essential for comparing systems on a common translational axis.

Therefore, the necessity of this review lies in bridging a persistent translation gap: despite rapid platform growth, clinically viable oral targeted therapies remain limited because performance depends on barrier heterogeneity, dynamic biointerfaces (mucus/protein corona), manufacturability, and safety under repeated dosing. This review examines the journey “from bench to bowel” by explicitly coupling clinical needs (e.g., IBD, GI cancers, peptic ulcer disease) with segment-specific barriers and the delivery mechanisms most suited to overcome them. We detail the biological barriers throughout the GI tract, analyze the core challenges in designing effective delivery platforms, and critically evaluate recent advances in nanocarrier-based systems, hydrogel technologies, and microscale delivery devices (Griffith et al., 2021). Furthermore, we elucidate the underlying mechanisms of passive, active, and stimuli-responsive targeting. Finally, we highlight how these strategies can enhance local efficacy while minimizing systemic toxicity, providing a barrier-guided roadmap for rational design and clinical translation of oral targeted therapies. Therefore, this review adopts a barrier-guided framework that systematically links GI region-specific physiological constraints to delivery challenges and the corresponding engineering strategies.

## 2 Barrier–challenge–solution map across GI segments

This section establishes the physiological foundation for the subsequent discussion by defining how region-specific GI barriers translate into distinct delivery challenges and solutions. The GI tract is a highly compartmentalized organ system optimized for digestion and absorption, comprising the oral cavity, esophagus, stomach, small intestine, and colon, each with distinct physicochemical and biological microenvironments6. While oral administration remains the most convenient and widely used route, effective therapy for GI diseases is limited by segment-specific barriers that impose predictable, region-dependent delivery challenges. Accordingly, in this section we adopt a standardized Barrier–Challenge–Solution organization for each GI segment: we first summarize the dominant local barriers (e.g., pH, enzymes, mucus architecture, epithelial permeability, immune/microbial factors), then specify the resulting obstacles to stability, retention, penetration, and controlled release, and finally highlight representative engineering solution classes that address them. This barrier-guided mapping framework is consolidated in Figure 1 and Table 1, enabling direct comparison of region-matched design logic across the GI tract. Representative mechanism-oriented delivery strategies for overcoming region-specific gastrointestinal barriers are summarized in Table 2.

**TABLE 2:** Mechanism-oriented summary of functional delivery strategies for overcoming region-specific gastrointestinal barrier.

Functional strategy	Primary GI region	Target barrier and challenges	Mechanisms	Diseases	Cargo type	Key limitation	Animal models	Ref.
Mucus-interacting strategies	Stomach (tumor)	Poor tumor penetration/distribution; liver uptake	<20 nm HER2-Fab star polymer improves penetration; SN-38 prodrug linker	HER2+ gastric cancer	SN-38	Linker release suboptimal; payload–carrier disconnect	NCI-N87 xenograft (SCID mice)	Sonzini et al. (2025)
Mucus-interacting strategies	Stomach (tumor mucosa)	Gastric mucus blocks tumor access; acid barrier	Motile urease + flagella *H. pylori* carries Ce6; mucus penetration + PDT/ICD	Gastric cancer	Ce6	Need post-therapy bacterial clearance; mild gastritis risk	Orthotopic MKN45-luc (BALB/c nude)	Zeng et al. (2024)
Mucus-interacting strategies	Stomach (mucosa)	Short residence; mucus barrier; acid instability	Alginate–chitosan core–shell mucoadhesive MPs; gastroretention; pH-biased release	Gastric ulcer	Puerarin	Chitosan adhesion drops > pH6.5; distal delivery limited	SD rats (ulcer); C57 mice (GI imaging)	Hou et al. (2014)
pH-responsive and enteric protection strategies	Stomach and duodenum	Acid/enzymes degradation; low local exposure	Enteric polymers protect acid-labile drugs; pH-triggered release (review)	Peptic ulcer disease	PPIs/antibiotics/etc (review)	Heterogeneous evidence; mainly preclinical	Rat ulcer models (e.g., ethanol)	Spósito et al. (2021)
pH-responsive and enteric protection strategies	Stomach (gastroretentive)	Rapid emptying; low antibiotic concentration	Porous Eudragit RS MPs (electrospray): floating and sustained MTZ release; prolonged gastric retention	*H. pylori* infection	Metronidazole	Higher conc. cytotoxicity; mucosal recovery lags	Rabbits (imaging) and mice (infection)	Hao et al. (2014)
Enzyme- and microbiota-triggered systems	Inflamed colon	Upper-GI stability; inflamed-site enrichment	Chi/Alg LBL protects + electrostatic inflamed-colon targeting; ROS triggers CO release; M2 polarization and NF-κB/p38 suppression	IBD/colitis	CO donor (Fe3(CO)12) in MPDA NPs	CO release poorly controllable; small-animal only	DSS-induced colitis mice (C57BL/6)	Zhang et al. (2023)
Enzyme- and microbiota-triggered systems	Colon (inflamed); systemic inflamed tissue	Acid/enzymes; mucus; epithelial barrier; site-specific release	PEG–HCCP–PBE peptide prodrug self-assembles NPs; ROS-triggered self-immolation releases native peptide	Colitis; acute lung injury	Anti-inflammatory peptides (KPV/Ac-QAW/IRW)	Trigger depends on pathological ROS; systemic distribution possible	DSS-induced colitis mice; ALI mice	Cheng et al. (2026)
Enzyme- and microbiota-triggered systems	Colon	Premature SSZ release; low azo-reductase; probiotic survival	Colon pH/β-glu degrades EudS-100/CS for release; spores germinate and provide azo reductase to activate SSZ (5-ASA)	IBD (UC/CD)	SSZ + *Clostridium butyricum* spores	Needs further clinical validation	DSS- and TNBS-induced colitis models	Zhao et al. (2026)
Redox/ROS/inflammation-responsive systems	Stomach (gastric retention)	Diosmin poor solubility/variable oral absorption; need mucus adhesion and retention	Chitosan coating increases positive zeta potential and mucoadhesion, prolonging gastric retention and sustained release, and alleviating NF-κB–related inflammation and mucosal injury	Ethanol-induced gastric ulcer	Diosmin (small molecule) in PLGA NPs (chitosan-coated)	High dose still used (100 mg/kg in rats); mainly preclinical evidence	Male Sprague–Dawley rats; 70% ethanol-induced ulcer	Abd El Hady et al. (2019)
Redox/ROS/inflammation-responsive systems	Colon (inflamed)	High ROS weakens phage therapy; needs GI protection/colon delivery	DNA origami ROS scavenging; Sg-phage lysis; enteric L100-55 release in intestine	IBD therapy; CRC prevention (Sg-linked)	Sg-specific phage + DNA nanopatch; Eudragit L100-55 coating	Phage–DNA conjugation/stability complexity; colon retention challenge	Male C57BL/6J DSS + Sg colitis; DSS + AOM + Sg CAC.	Zhao Y. et al. (2025)
Redox/ROS/inflammation-responsive systems	Colon (inflamed)	Gastric juice; poor lesion targeting; rapid metabolism/clearance	Hp microalgae + Pt nanozyme scavenges ROS; electrostatic colon targeting/retention (PAA); barrier repair (tight junctions)	IBD/colitis (DSS)	Hp@CS-PNAs@PAA biohybrid microrobot (astaxanthin + Pt nanozyme)	Scale-up/standardization; cost and storage stability for long-term use	7-week female BALB/c DSS colitis	Yang et al. (2025)
Epithelial transport enhancement and absorption boosting	Small intestine	Mucus + tight junctions limit uptake	Reversible TJ modulation (integrin/ZO-1); mucus-penetrating coatings; pH-responsive oral carrier	Diabetes (oral insulin)	Protein/biologic	TJ-safety window; mucus trapping risk	Caco-2; diabetic mice	Finbloom et al. (2020)
Epithelial transport enhancement and absorption boosting	Colon; colonic mucus/epithelium	Acid/enzymes/nucleases; mucus trapping; epithelial barrier; immune clearance	HA–TMAO zwitterionic coat for mucus penetration; OCTN2-mediated epithelial uptake/transcytosis; CRISPR KO of TRAP1 triggers CypD–mPTP opening and boosts immunogenic necrosis/ICD	Colorectal cancer (incl. chemoresistant; spontaneous CRC)	Oral CRISPR–Cas9 plasmid DNA (sgTRAP1/Cas9)	Editing efficiency moderate; translational/off-target validation needed	CT26-Luc orthotopic caecum CRC; chemoresistant CRC; ApcMin/+ spontaneous CRC; CRC organoids	Zhao K. et al. (2025)
Device-assisted and microscale barrier-bypass strategies	Stomach (HER2+ gastric tumor)	Poor intratumoral penetration/distribution of NPs	HER2-Fab–decorated <20 nm star polymer enhances HER2 binding/uptake and tumor penetration/retention	HER2+ gastric cancer	SN-38–conjugated polysarcosine star polymer + HER2-Fab	Payload release can be rapid, reducing late-time differences	Gastric cancer xenograft in mice	Lee and Chang (2021)

### 2.1 Oral cavity

The oral cavity provides a near-neutral pH (6.6–7.1) and rich vascularization, yet drug delivery is constrained by extremely short transit time and continuous salivary clearance, together with degradative enzymes such as amylase and the intrinsically low permeability of buccal mucosa (typically suitable only for small, lipophilic molecules) (Lou et al., 2023). Because residence time is extremely limited, the primary challenge is insufficient exposure for absorption, and salivary washout further reduces local concentration, thereby necessitating retention-enhancing solutions such as mucoadhesive designs to prolong mucosal contact (Bobo et al., 2016). In parallel, enzymatic activity directly translates into payload instability, so enzyme-inhibition/protective strategies must be incorporated to preserve drug integrity within the short window (Durán-Lobato et al., 2020a; Han et al., 2024). Finally, low buccal permeability limits macromolecule and particle transport, which motivates the use of permeation-enhancing/penetration-facilitating approaches to improve epithelial entry (Miao et al., 2021), while maintaining robust biocompatibility for repeated mucosal contact (Ren et al., 2023).

### 2.2 Esophagus

The esophagus primarily functions as a conduit transporting formulations to the stomach via peristalsis; although a thin mucus layer protects the lining, limited surface area, minimal blood supply, and short transit time constrain meaningful absorption (Batchelor, 2005). This rapid, peristaltic transit creates a direct cause-and-effect link: short residence time leads to poor localization and weak mucosal interaction, so successful esophageal therapy (when needed) depends first on retention/localization-focused designs rather than systemic absorption goals. Because epithelial transport is inherently limited, strategies that emphasize rapid positioning and local protection (e.g., protective coatings) become more practical than attempting high-efficiency uptake (Bobo et al., 2016), and given the risk of irritation under repeated exposure, safety/tolerability must be treated as a design constraint rather than a downstream consideration (Ren et al., 2023).

### 2.3 Stomach

The stomach presents the most severe early barrier for oral delivery due to intense acidity (pH 1.0–4.0) (Durán-Lobato et al., 2020b) and pepsin-driven proteolysis, which readily denature or digest acid-labile biologics such as insulin. Here the barrier-to-challenge mapping is explicit: low pH and proteases directly cause chemical/enzymatic degradation, which in turn demands protective strategies that stabilize payloads before they can reach the intended site of action (Durán-Lobato et al., 2020a; Han et al., 2024). Beyond chemical degradation, the gastric mucin–bicarbonate barrier and epithelial tight junctions further restrict access to absorptive surfaces and limit transport (Durán-Lobato et al., 2020b). Therefore, diffusion/penetration limits imposed by mucus and epithelial sealing must be addressed without compromising mucosal safety (Miao et al., 2021; Ren et al., 2023), and the “premature release” problem in the stomach must be converted into “triggered release” downstream through enteric or pH-responsive control (Zhang LZ. et al., 2024). Clinically, these constraints are reflected in “protected + absorption-aided” formulations: oral semaglutide is co-formulated with the permeation enhancer SNAC to enable oral peptide uptake (Aroda et al., 2022), while enteric-coated oral insulin ORMD-0801 incorporates protease inhibition and permeability-enhancing excipients to reduce gastric loss and improve downstream absorption (Eldor et al., 2023).

### 2.4 Small intestine

After surviving the stomach, the small intestine becomes the principal absorption site, offering vast villus/microvillus surface area and relatively prolonged transit time (∼10 h) under near-neutral pH (5.9–7.8) (Koziolek et al., 2015). However, delivery here faces tightly coupled hurdles: pancreatic enzymes rapidly degrade proteins and nucleic acids; a dense mucin glycoprotein layer hinders diffusion; epithelial tight junctions restrict paracellular passage; many biologics have unfavorable physicochemical properties for passive transcellular transport; and efflux transporters can pump absorbed molecules back into the lumen, all while mucosal immune surveillance can recognize exogenous antigens (Coffey et al., 2021; Lim et al., 2013). Consequently, each dominant barrier corresponds to a predictable failure mode: enzymes drive payload loss, mucus thickness drives diffusion limitation and entrapment, tight junctions restrict permeability, efflux lowers net uptake, and immune surveillance increases clearance risk, so effective systems must integrate multi-layer solutions rather than optimizing a single parameter (Durán-Lobato et al., 2020a; Han et al., 2024). Specifically, mucus entrapment and epithelial restriction motivate mucus-penetrating surface engineering (e.g., PEGylation and related designs) to improve traversal and access to absorptive epithelium,^13^while spatiotemporally correct release must be coordinated at absorptive sites rather than occurring upstream (Zhang LZ. et al., 2024). When biochemical and biophysical barriers remain dominant, device-assisted “barrier bypass” provides a direct solution, exemplified by an ingestible luminal unfolding microneedle injector that achieved oral systemic delivery of macromolecules with bioavailability exceeding 10% of subcutaneous injection over 4 h (Abramson et al., 2019). Throughout, because the small intestine is immunologically active, biocompatibility and immune safety are not optional add-ons but core design constraints.

### 2.5 Colon

Downstream, the colon provides near-neutral pH (6–6.7) and the longest residence time (∼20 h), which can favor localized action and sustained exposure (3D printing technologies, 2025) Yet this advantage is counterbalanced by a relatively thick mucus barrier and a highly diverse microbiota that can degrade therapeutics—creating both a stability risk and an opportunity for site-specific activation (Amidon et al., 2015). Thus, the key causal chain differs from the upper GI tract: prolonged residence supports local therapy, but thick mucus reduces access to the epithelium and microbial metabolism can inactivate drugs before they act, so colon targeting must jointly solve retention, mucus traversal, and colon-restricted release. Accordingly, successful colon targeting emphasizes precise localization/retention and controlled, colon-restricted release to maximize local efficacy while minimizing upstream absorption (Bobo et al., 2016; Zhang LZ. et al., 2024), alongside mucus-penetrating or mucoadhesive strategies combined with protection against microbiota-associated degradation until arrival at the action site. These principles align with classic colon-directed therapies such as 5-ASA, whose efficacy depends on delivering drug to the colon while limiting absorption elsewhere (Safdi and Sandborn, 2008), often achieved via pH-dependent coatings, time-dependent release systems, or microbiota-activated prodrug strategies (e.g., azo-bond cleavage) that convert microbial activity from a degradation risk into a release trigger (Malayandi et al., 2014; Kiilerich et al., 2025; Wang A. et al., 2024). More recent examples further connect barrier logic to engineering solutions, including microbiota-involved delivery designs for colorectal cancer therapy and ROS-responsive approaches that form an “artificial mucus layer” *in situ* to modulate the inflamed colonic microenvironment while improving local retention and barrier function.

### 2.6 Summary: a region-to-challenge design rule for GI targeting

Collectively, the GI tract imposes region-dependent barrier constellations, meaning that successful targeted systems must solve different subsets of the same core challenges depending on the intended site of action. In practical design terms, this implies a region-matched priority order: upper GI emphasizes protection against acid/proteases and premature release, the small intestine emphasizes integrated mucus/epithelium traversal plus absorption constraints, and the colon emphasizes mucus–microbiota interactions coupled with colon-restricted activation, while maintaining strict safety and long-term tolerability.

## 3 Functional delivery mechanisms to address region-specific GI barriers

**FIGURE 2:**
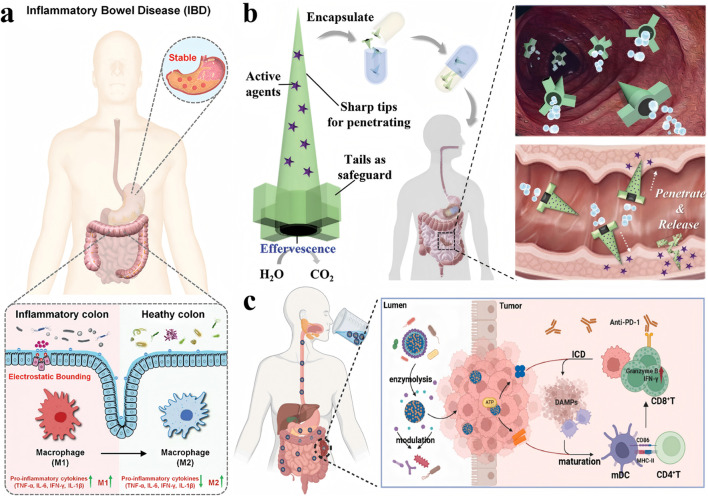
Representative studies illustrating key functional delivery mechanisms in Section 3. **(a)** Schematic illustration of a redox/ROS/inflammation-responsive system for IBD therapy. (Panel (a) adapted from Yu et al., *Advanced Materials* 2023, 35, 2304967. © 2023 The Authors. Published by Wiley-VCH GmbH. Open access.) (Yu et al., 2023). **(b)** Schematic illustration of a device-assisted/microscale barrier-bypass strategy for oral macromolecule delivery. **(b)** adapted from Cai et al., *Advanced Materials* 2023, 35, 2210679. © 2023 The Authors. Published by Wiley-VCH GmbH. Open access.) (Cai et al., 2023). **(c)** Schematic illustration of an enzyme-/microbiota-triggered activation strategy for oral colorectal cancer therapy. **(c)** reprinted from Sun et al., *Materials Today Bio* 2025, 35, 102565. © 2025 (Sun et al., 2025) The Authors. Published by Elsevier Ltd. This is an open access article under the CC BY-NC-ND 4.0 license) (Fleseriu et al., 2022).

In the following sections, delivery systems are discussed in the context of the specific barriers they are designed to overcome rather than by material classification alone. Section 2 establishes that the dominant obstacles to oral and local GI therapy are not uniform but instead emerge from region-dependent barrier constellations (Durán-Lobato et al., 2020b; 3D printing technologies, 2025). In practice, a single “platform” (nanoparticles, hydrogels, or microscale devices) can implement multiple mechanisms; conversely, a single mechanism (e.g., mucus penetration) can be achieved using diverse platforms. Therefore, we reorganize the discussion around six recurring functional strategy families that directly map to the barrier–challenge–solution axis summarized in Figure 1 and Table 1: mucus-interacting strategies; pH-responsive/enteric protection; enzyme- and microbiota-triggered activation; redox/ROS/inflammation-responsive systems; epithelial transport and absorption boosting; and device-assisted/microscale barrier bypass (Malayandi et al., 2014; Kiilerich et al., 2025; Wang A. et al., 2024). Representative examples of three major mechanism-oriented strategy families are further illustrated in Figure 2.

A mechanism-oriented taxonomy also enables critical benchmarking across studies. Many preclinical GI delivery reports demonstrate “targeting” via improved local accumulation or histological benefit, yet translation remains limited because performance depends on heterogeneous mucus architecture, dynamic biointerfaces (including protein corona formation in GI fluids), individual variability in pH and microbiota, and long-term safety under repeated dosing (Guo et al., 2021; Aroda et al., 2022; Wang A. et al., 2024). Furthermore, successful systems must satisfy manufacturability constraints (batch-to-batch reproducibility, scalable surface modification, sterilization, and cost) and demonstrate robust pharmacokinetics and safety in models that better approximate human physiology (Hou et al., 2014). The sections below therefore emphasize (i) the causal linkage between barrier and failure mode, (ii) how each mechanism addresses that failure mode, (iii) representative examples drawn from current research, and (iv) translational gaps—particularly scalability, safety, and comparative efficacy—so that readers can compare solutions on a common translational axis rather than by carrier type alone (Zhu et al., 2024; Griffith et al., 2021).

### 3.1 Mucus-interacting strategies

Across the stomach–intestine–colon axis, the mucus gel functions as a selective, dynamic filter; because mucin networks can immobilize particulates through hydrophobic and electrostatic interactions, increased mucus thickness and viscosity directly translate into diffusion limitation and delayed epithelial access, ultimately lowering effective local dose at the mucosa (Miao et al., 2021; Wang A. et al., 2024). As a result, mucus-interacting designs typically follow two contrasting logics: mucoadhesive strategies aim to increase local residence by anchoring to mucus (useful for local depot formation but potentially prone to “stuck-in-mucus” failure), whereas mucus-penetrating strategies minimize adhesive interactions to traverse mucus and reach the epithelial surface for absorption or cell uptake (Miao et al., 2021). Mechanistically, mucus penetration is often promoted by reducing non-specific binding (e.g., hydrophilic shielding and charge neutralization) and by optimizing size so that carriers can navigate mucus mesh while avoiding rapid clearance (Miao et al., 2021). Importantly, once a carrier enters GI fluids, adsorption of luminal biomolecules can reshape its surface identity (“protein corona”), which can shift mucus interactions and partially explain why mucus-optimized designs may show variable *in vivo* performance (Lundquist and Artursson, 2016). Representative studies illustrate how these principles are implemented in disease models: Rampado et al. engineered lipid nanoparticles with fine-tuned composition that showed enhanced colon targeting as a platform for mRNA therapeutics, supporting the idea that surface/composition engineering can bias transport and localization in inflamed colon environments (Hao et al., 2014). In another conceptually related direction, Zhang et al. reported an oral ROS-responsive system that forms an “artificial mucus layer” *in situ* for IBD treatment, highlighting that mucus can be not only a barrier to traverse but also a targetable interface to modulate for therapeutic benefit. From a translational perspective, mucus-interacting strategies are attractive because they are broadly applicable, but their performance can be sensitive to patient-to-patient mucus variability and to the evolving biointerface in GI fluids.

### 3.2 pH-responsive and enteric protection strategies

The GI tract imposes steep pH gradients; because gastric acidity (pH ∼1–4) can denature biologics and destabilize acid-labile drugs, low pH directly causes payload loss before the carrier reaches the intended intestinal site, making “survive first, release later” a central design requirement (Durán-Lobato et al., 2020a; Han et al., 2024; Batchelor, 2005). Consequently, pH-responsive strategies typically pursue either (i) enteric protection, where coatings remain intact in the stomach and dissolve/swollen in higher-pH regions, or (ii) pH-triggered carrier transformation, where pH changes drive bond cleavage, swelling, or permeability shifts to enable controlled release at downstream sites (She et al., 2025; Zeng et al., 2024). This logic underpins colon-directed approaches where pH-dependent release helps maximize colonic exposure while limiting upstream absorption (Malayandi et al., 2014). Concrete examples from the literature show how pH is harnessed to convert a destructive barrier into a spatial trigger: Gu et al. developed pH-sensitive liposomes co-encapsulating docetaxel and pemetrexed, aiming to exploit pH-linked behavior for tumor-targeted delivery with reduced off-target toxicity (Coco et al., 2013). Clinically anchored cases further reinforce the “protection plus enabling” principle: oral semaglutide was developed by co-formulating semaglutide with SNAC, illustrating that even when gastric survival is improved, uptake still benefits from mechanisms that assist epithelial transport (Durán-Lobato et al., 2020b). Similarly, the enteric-coated oral insulin formulation ORMD-0801 combines protease inhibition and permeability-enhancing excipients to reduce gastric loss and improve downstream absorption. From a translational standpoint, pH-triggered designs are conceptually simple and scalable in many cases, but their reliability must contend with inter-individual pH variability and food effects, which can shift release thresholds (Aroda et al., 2022).

### 3.3 Enzyme- and microbiota-triggered systems

Enzymatic degradation and microbial metabolism are pervasive in the GI tract; because digestive enzymes in the small intestine rapidly cleave proteins/nucleic acids and colonic microbiota can metabolize drugs, these biochemical factors directly translate into premature inactivation or off-target loss, but they also provide region-informative triggers that can be harnessed for site-specific activation (Abramson et al., 2019), (Safdi and Sandborn, 2008; Malayandi et al., 2014). Therefore, enzyme- and microbiota-triggered systems are designed to remain stable during upper-GI transit and then undergo cleavage/degradation in response to targeted enzymes (host or bacterial), enabling colon-restricted or disease-restricted release. Classic colon targeting exemplifies this concept through microbiota-activated prodrugs, which convert colonic bacterial activity into a reliable release mechanism for drugs such as 5-ASA (3D printing technologies, 2025; Amidon et al., 2015). More recent carrier-based implementations extend this logic to advanced therapeutics: A representative microbiota-triggered activation strategy is shown in Figure 2c. Shen et al. reported a hierarchically targetable polysaccharide-coated solid lipid nanoparticle system, where colon-associated bacterial dextranase functions as a trigger, thereby linking a specific microbial enzyme cue to colonic drug release and local therapy (Cai et al., 2023). Multi-trigger designs further illustrate how microbial cues can be combined with disease chemistry to improve specificity; Sun et al. engineered dual-responsive microspheres for CRC treatment that leveraged colorectal β-mannanase alongside tumor ROS to achieve spatiotemporally controlled release and therapeutic benefit (Li et al., 2024). In hydrogel-based approaches, Tang et al. developed an oral pH/enzyme-sensitive hydrogel system (Kae/CMCHD@RNs) for ulcerative colitis that enabled sustained colon-specific release and macrophage-targeted delivery, demonstrating how enzyme responsiveness can be embedded within a protective matrix for distal-gut targeting (Folate decorated multifunctional biodegradable nanoparticles, 2026). From a translational standpoint, enzyme/microbiota-triggered strategies are powerful for colon specificity, but their robustness can be influenced by microbiota heterogeneity and external perturbations (diet/antibiotics).

### 3.4 Redox/ROS/inflammation-responsive systems

Inflammatory GI diseases commonly exhibit elevated ROS and oxidative stress; because inflamed mucosa and tumor microenvironments produce higher ROS/RNS levels than healthy tissue, redox gradients and ROS become disease-associated triggers that can directly activate carriers at lesion sites, enabling selective release or local microenvironment modulation. In this strategy family, carriers respond to ROS/redox cues *via* bond cleavage (e.g., thioketal-like linkers), matrix disassembly, or catalytic activity that simultaneously scavenges harmful species and restores barrier function (Zhou et al., 2025; She et al., 2025). A representative schematic of this inflammation-localized redox/ROS-responsive strategy is provided in Figure 2a. Representative examples from your manuscript demonstrate both “triggered release” and “triggered therapy” modes. Kim et al. developed Aurozyme, in which AuNPs coated with glycol chitosan/GL shifted catalytic behavior toward catalase-like activity, enabling ROS/RNS and DAMP scavenging and thereby alleviating colitis-associated inflammatory stress (Cheng et al., 2026). At the microscale, Zhao et al. created inflammatory microenvironment-responsive microspheres for IBD that targeted inflamed colon sites and responsively released immunomodulators (MXene nano immunomodulators) and L-arginine (converted to nitric oxide), illustrating how inflammatory cues can be coupled to local immunoregulatory payloads (Magnetic Nanoparticles for Stimuli-Responsive, 2025). In addition, Zhang et al. reported a ROS-responsive oral system that forms an “artificial mucus layer,” demonstrating that ROS triggering can drive *in situ* barrier reinforcement and improved local retention in colitis models. Taken together, these studies reflect a consistent causal logic: inflammation elevates ROS; elevated ROS activates carriers; activation concentrates therapy locally while limiting systemic exposure. From a translational standpoint, this class is appealing for IBD and inflamed tumors, but trigger intensity can vary with disease stage, so dose–response relationships should be characterized across severity conditions.

### 3.5 Epithelial transport enhancement and absorption boosting

Even when carriers survive pH/enzymes and negotiate mucus, the epithelium remains a major bottleneck; because tight junctions restrict paracellular passage and many biologics have poor transcellular permeability (with efflux and immune surveillance further reducing net uptake), epithelial transport limitations directly cause low bioavailability and weak lesion penetration, especially for macromolecules (Koziolek et al., 2015). Thus, absorption boosting strategies aim to convert an epithelial barrier into a controllable interface by using permeation enhancers, transient modulation of epithelial transport pathways, or alternative uptake routes such as intestinal lymphatic transport (Miao et al., 2021). Clinically, oral semaglutide provides a clear example of this mechanism: Aroda et al. summarized how SNAC enabled oral peptide uptake, demonstrating that transport enhancement can be the enabling step that turns a protected peptide into an effective oral medicine (Durán-Lobato et al., 2020b). Similarly, Eldor et al. reported a randomized study of oral insulin (ORMD-0801) where the formulation incorporated protease inhibition and permeability-enhancing excipients, reflecting the same causal chain: reduce upstream loss, then actively assist epithelial entry to improve downstream exposure. At the design-principle level, Miao et al. discussed engineering nano- and microparticles as oral delivery vehicles to promote intestinal lymphatic drug transport, highlighting a route that can help bypass first-pass constraints and potentially improve systemic exposure for certain payload classes. In practice, these approaches must balance efficacy with mucosal safety; because repeated epithelial perturbation may affect barrier integrity and immune recognition, tolerability under chronic dosing remains a key consideration, even if discussed briefly.

### 3.6 Device-assisted and microscale barrier-bypass strategies

When biochemical protection and nanoscale engineering remain insufficient, physical or microscale approaches offer direct solutions; because devices can bypass mucus and epithelial transport constraints by mechanically placing payloads into or across the mucosa, they can convert a low-permeability epithelium into a deliverable target surface, particularly for biologics (Hao et al., 2022; Repetitive drug delivery using light-activated, 2025; Stimuli Responsive Nanosonosensitizers for Sonodynamic, 2025). A representative device-assisted barrier-bypass strategy is illustrated in Figure 2b. Representative examples in your manuscript illustrate multiple implementations of barrier bypass. Abramson et al. developed an ingestible “luminal unfolding microneedle injector” for oral delivery of macromolecules; using insulin, the device achieved rapid uptake and systemic bioavailability exceeding 10% of subcutaneous injection over 4 h, demonstrating that physical epithelial penetration can overcome absorption bottlenecks that chemistry alone struggles to address (Magnetic Nanoparticles for Stimuli-Responsive, 2025). In microrobotic systems, Gao et al. reported a bubble-driven gastric dissolving microrobot that self-propelled in gastric acid, embedded in the stomach mucosa, and autonomously released payload, directly linking the stomach’s acidic barrier to a propulsion-and-localization mechanism (Lai et al., 2009). Wang et al. further proposed a multichambered tubular micromotor with a zinc-propelled backend and a gelatin frontend capped by pH-responsive protection, which enhanced gastric tissue distribution/retention by overcoming acidic and motility-related constraints (Niebel et al., 2012). Microscale carriers can also provide sustained local exposure; for example, Thalia S.A. Lemos et al. developed magnetic chitosan microspheres where incorporated magnetite enabled magnetic-field-enhanced release, illustrating how external fields can be used to modulate release kinetics after localization (Malayandi et al., 2014; Kiilerich et al., 2025; Wang A. et al., 2024). From a translational standpoint, these systems are promising where “delivery physics” is required, but practical deployment depends on safety (mucosal irritation), reproducibility, and cost—here we note these considerations without expanding in detail.

## 4 Mechanism of targeting drug delivery

**FIGURE 3:**
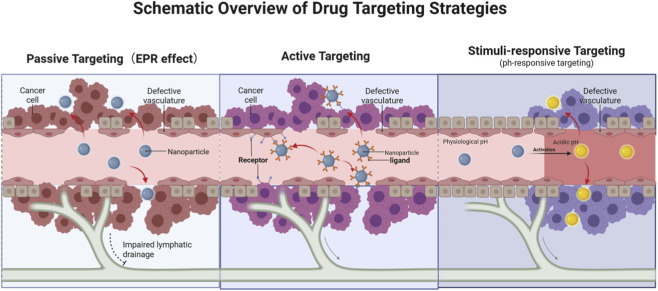
Schematic overview of three major drug-targeting mechanisms: passive targeting, active targeting, and stimuli-responsive targeting (take ph-responsive targeting as an example).

These targeting mechanisms represent functional solutions to the biological barriers described above and provide a common mechanistic basis for comparing different delivery platforms. The core principles of passive, active, and stimuli-responsive targeting are schematically illustrated in Figure 3. Notably, oral octreotide capsules have reached phase 3 evaluation: in a randomized trial, oral octreotide was non-inferior to injectable somatostatin receptor ligands for maintaining biochemical response in acromegaly, with 91% (50/55) of patients maintaining response during the randomized phase (Fleseriu et al., 2022). In the MPOWERED open-label extension, biochemical response was maintained in 89.7% at year 1, 87.8% at year 2, and 93.5% at year 3 among those entering each year as responders, with no new or unexpected safety signals (Fleseriu et al., 2023).

### 4.1 Passive targeting mechanism

The mechanism of passive targeting, which serves as the foundation for both active and stimuli-responsive targeting strategies, is primarily attributed to the enhanced permeability and retention (EPR) effect. First reported by Matsumura and Maeda in 1986 (Matsumura and Maeda, 1986), the EPR effect is classically exploited in tumor targeting. Solid tumors possess defective vasculature with enhanced permeability and poor lymphatic drainage, allowing macromolecules and nanoparticles to extravasate and remain in tumor tissue for prolonged periods (Matsumura and Maeda, 1986; Fang et al., 2011). However, this classical tumor EPR framework should not be directly transplanted to GI inflammation or GI tumors (Cepero et al., 2022). In the GI tract, passive accumulation is additionally shaped by the mucus barrier, epithelial tight junctions, rapid luminal clearance, regional pH and enzyme gradients, and marked inter-individual variability, all of which are not core defining features of the classical vascular EPR model in solid tumors (Yang et al., 2026; Zhu et al., 2021). Accordingly, for inflamed intestinal lesions, it is more rigorous to describe the phenomenon as an inflammation-associated permeability/retention process or an EPR-like effect, rather than assuming full equivalence to the classical tumor EPR effect (Yang et al., 2026; Wan et al., 2025). Recent work has even used the term epithelial enhanced permeability and retention (eEPR) to emphasize this GI-specific context (Yang et al., 2026). For GI tumors, passive accumulation may still benefit from permeability-related retention, but dense extracellular matrix, elevated interstitial fluid pressure, hypoxia, mucus coverage, and heterogeneous local microenvironments can substantially restrict nanoparticle penetration and distribution, making the magnitude and predictability of this effect more variable than the simplified classical EPR description suggests (Cepero et al., 2022). Therefore, passive targeting in the GI tract should be interpreted as a barrier-conditioned process jointly governed by vascular permeability, epithelial integrity, mucus interaction, and local pathological heterogeneity (Zhu et al., 2021).

Passive targeted drug delivery is mainly achieved through delivery systems, especially nanoparticles and liposomes. Gu et al. (2023) developed pH-sensitive liposomes via thin-film hydration to co-encapsulate hydrophobic docetaxel and hydrophilic pemetrexed, enabling tumor-targeted delivery through passive accumulation (validated by cellular/biodistribution studies) with reduced off-target toxicity. pH and glutathione-responsive (2025) developed pH/GSH dual-responsive PMDC NPs (pHCT74/MOF-5@DHA&CORM-401) for targeted colorectal cancer therapy. These nanoparticles leveraged EPR effects mediated passive targeting and pHCT74 peptide-mediated active targeting toward α-enolase-overexpressing tumors. Li et al. (2019) developed a self-assembled, oxidation-degradable Janus-prodrug nanoparticle (B-ATK-T NP). Upon oral administration in IBD mice, B-ATK-T NP demonstrated significant passive accumulation in the inflamed colon, achieving dramatically elevated local drug concentrations while minimizing systemic exposure. This enhanced site-specific delivery contributed to superior anti-inflammatory and antioxidative efficacy over free drugs. Furthermore, passive targeting have also shown promising abilities in disease cure with other chemotherapeutics, such as hydroxychloroquine (Elshami et al., 2023), naringenin (Lung cancer cells, 2025), and cromolyn (Motawi et al., 2017). But in general, in passive targeted therapy research, the primary focus lies in engineering drug carriers to prolong systemic circulation and achieve intended site-specific accumulation. Key design parameters—including size (Ren et al., 2019), rigidity (Gurnani et al., 2022), and surface PEGylation (Liu et al., 2021) of liposomal delivery systems—critically govern their biodistribution and therapeutic efficacy. For example, a study on the targeting of rheumatoid arthritis (RA) clarified the effect of size on liposome delivery. It showed egg yolk lecithin (EPC) liposomes with a size of 100 nm had a longer blood circulation time of 12.85 h than liposomes with a size of 70 nm, 200 nm or 350 nm in healthy mice (Ren et al., 2019).

### 4.2 Active targeting mechanism

Active targeting functionalizes drug carriers with ligands (e.g., antibodies/peptides) to bind disease-specific targets. This ligand-receptor interaction enables precise molecular recognition and site-selective accumulation, enhancing local drug concentration and therapeutic efficacy. Implementation strategies for active targeting primarily involve ligand conjugation to carrier platforms, including liposomes (Paclitaxel-loaded ginsenoside Rg3, 2025), monoclonal antibodies, antibody-drug conjugates (ADCs) (Breaking barriers in triple negative breast cancer, 2026), and engineered nanocarriers (NIH, 2025; Folate decorated multifunctional biodegradable nanoparticles, 2026).

The active targeting mechanism has a wide range of applications in GI diseases. For example, AhR agonists developed for the treatment of IBD have entered phase I clinical stage. Hang et al. (2025) found that napabucasin can inhibit *Helicobacter pylori* through an active targeting mechanism. When studying the treatment of colorectal cancer, *Hiroshi* (Sootome et al., 2020) found that preclinical profile of futibatinib, a structurally novel, irreversible FGFR1-4 inhibitor which exhibited potent, selective growth inhibition of several tumor cell lines including gastric, harboring various FGFR genomic aberrations. Chen et al. (2021) constructed a hybrid nanoplatform by integrating mesoporous silica nanoparticles, a supported lipid bilayer, and cetuximab to effectively encapsulate 5-fluorouracil (5-FU) and enable its selective delivery against colorectal cancer (CRC) cells. They also found that the modification of cetuximab onto nanoplatforms without drug loading can significantly inhibit the migration and invasion of CRC cells through suppressing the epidermal growth factor receptor (EGFR)-associated signaling pathway.

Active targeting also faces challenges: off-target effects from low antigen expression in healthy tissues, spatial heterogeneity in lesions, patient variability affecting predictability, costly ligand conjugation requiring rigorous validation, and stability issues. By comparison, passive targeting faces a different set of bottlenecks: it depends strongly on lesion permeability, mucus architecture, luminal transit, and carrier physicochemical properties, so disease-site accumulation is often heterogeneous and insufficiently specific. In practice, improving passive targeting requires better control over size, rigidity, and surface shielding, together with region-matched release and retention design rather than relying on EPR-like accumulation alone (Ren et al., 2019; Gurnani et al., 2022). Stimuli-responsive targeting offers higher conditional selectivity, but its performance is constrained by fluctuating trigger intensity, possible premature activation, limited loading capacity, and more demanding material and manufacturing requirements; future strategies should therefore favor simplified architectures, quantifiable trigger thresholds, and validation in models that better capture human GI heterogeneity (Rezaei et al., 2022; Guo et al., 2025).

### 4.3 Stimuli-responsive targeting mechanism

Stimuli-responsive targeting uses drug carriers that change structure upon encountering specific GI triggers—like pH shifts, enzymes, or gut microbiota—to release therapeutics precisely at diseased sites. This enhances local drug concentration while minimizing systemic exposure, critical for treating IBD, colon cancer, and infections with reduced side effects. Upon reaching target sites or encountering specific stimuli, stimuli-responsive targeting systems undergo structural/property transformations—such as bond cleavage, phase transitions, or morphological shifts—triggering precision drug release. Responsive polymers can be activated by endogenous cues (e.g., Ph (HIF-2α, 2025; pH-sensitive multi-drug, 2025), redox (Hao et al., 2022), enzyme) or exogenous inputs (e.g., light (Repetitive drug delivery using light-activated, 2025), ultrasound (Stimuli Responsive Nanosonosensitizers for Sonodynamic, 2025), magnetism (Magnetic Nanoparticles for Stimuli-Responsive, 2025), generating therapeutic effects including ROS production and thermal ablation.

**FIGURE 4:**
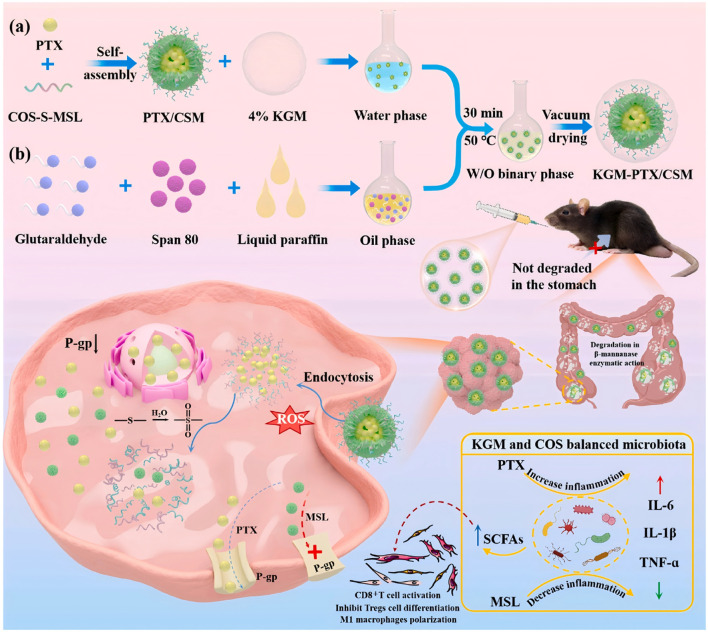
Schematic illustration of the preparation and therapeutic mechanism of the KGM‐PTX/CSM delivery system. **(a)** Aqueous‐phase preparation route of PTX/CSM and KGM. **(b)** Oil‐phase preparation route using glutaraldehyde, Span 80, and liquid paraffin. The water and oil phases are combined to form the W/O binary phase and then vacuum‐dried to obtain KGM‐PTX/CSM. The lower panel shows the intestinal degradation, cellular uptake, drug release, P‐gp inhibition, immune regulation, and microbiota/inflammatory modulation of the system. (Reprinted from Wang et al. Materials Today Bio, Vol 30, “Advanced Fe_3_O_4_@CS nanocomposites for duodenal cancer therapy”, Copyright (2025), with permission from Elsevier under CC BY 4.0 license. DOI:10.1016/j.mtbio.2025.101920).

Compared with single-responsive systems, dual-responsive platforms may reduce premature activation and improve spatiotemporal precision by requiring two disease-associated cues. However, this gain in selectivity is not universal; single-responsive systems are often more straightforward in mechanism, manufacturing, and quality control, and may remain preferable when one dominant trigger is sufficiently stable and disease-relevant. In addition to conventional single stimuli responsive targeted delivery, there are also multiple stimuli responsive targeted delivery. Sun et al. (2025) engineered dual-responsive KGM-PTX/CSM microspheres for CRC treatment. This oral system leveraged colorectal β-mannanase and tumor ROS for spatiotemporally controlled drug release. As is seen in Figure 4. Sun et al. demonstrated that ROS-triggered micelle disintegration and β-mannanase-triggered KGM degradation significantly inhibited tumor growth, modulated immunity, and restored gut microbiota.

Although promising, stimuli-responsive drug delivery systems for the gastrointestinal tract face severe challenges. The core issue lies in the extreme complexity of the internal digestive environment (e.g., dynamic pH, enzymes, mucus layer, and individual variations), which makes reliable triggering of exogenous stimuli and precise spatiotemporal drug release difficult. Furthermore, these systems must overcome the robust mucus barrier to achieve effective adhesion and retention at the target site (Rezaei et al., 2022), while also contending with limited drug-loading capacity. For dual-responsive platforms, these problems are further amplified because the system must maintain stable integration of two responsive modules during fabrication, storage, upper-GI transit, and arrival at the lesion site. As a result, dual-responsive systems may suffer from greater batch-to-batch variability, trigger-order uncertainty, and lower *in vivo* predictability than single-responsive designs, particularly when pH, ROS, enzymes, or microbiota differ across patients, disease stages, and intestinal segments. The intricate material synthesis and processing techniques render large-scale production challenging and costly, significantly hindering clinical translation (Guo et al., 2025). Future optimization should therefore emphasize simplified formulations, clearly defined trigger thresholds, sequential rather than merely additive responsiveness, and more human-relevant models for evaluating release reliability, stability, and safety. Additionally, existing animal models fail to fully replicate human physiology, resulting in uncertainties in preclinical safety and efficacy evaluations.

## 5 Applications of target delivery system in gastrointestinal diseases

### 5.1 IBD

IBD, encompassing Crohn’s disease and ulcerative colitis, is a chronic immune-mediated gastrointestinal disorder driven by genetic susceptibility, environmental factors, altered gut microbiota, and dysregulated mucosal immunity (Lee and Chang, 2021; Ni et al., 2017; Fiocchi, 2016). Conventional oral therapeutics, including aminosalicylates, corticosteroids, immunomodulators, and biologics like anti-TNF-alpha antibodies, often exhibit suboptimal therapeutic outcomes due to inadequate drug concentrations at inflamed sites and systemic absorption-related adverse effects (Wan et al., 2025). Consequently, targeted drug delivery strategies are essential to enhance local efficacy while minimizing systemic exposure and toxicity.

Initial efforts focused on exploiting colonic physiology for targeting due to the predominant colonic involvement in IBD pathogenesis. These included pH-sensitive coatings designed to dissolve at the elevated pH of the distal intestine or colon, time-dependent systems releasing drug after a lag time calibrated for colonic arrival, and prodrug strategies reliant on microbial activation (e.g., azo bond-conjugated 5-ASA) (Malayandi et al., 2014; Kiilerich et al., 2025; Wang A. et al., 2024). Building upon this foundation, advanced Nano-Delivery Systems (NDS) now offer enhanced precision. Size-based targeting in inflamed colon should be understood as leveraging an inflammation-associated permeability/retention phenomenon or EPR-like accumulation rather than the classical solid-tumor EPR effect; in IBD, local accumulation is additionally influenced by mucus properties, epithelial injury, immune-cell infiltration, microbiota-related changes, and lesion heterogeneity (Zhou et al., 2018; Zhu et al., 2021; Wan et al., 2025). Size-based targeting leverages the Enhanced Permeability and Retention (EPR) effect in inflamed tissue, where smaller nanoparticles (NPs) achieve prolonged colonic residence and increased immune cell uptake, primarily dependent on size and utilizing polymers like PLGA, PCL, PEG, and polystyrene (Xiao and Merlin, 2012; Lamprecht et al., 2001a; Collnot et al., 2012; Lamprecht et al., 2001b; Pichai and Ferguson, 2012; Lb et al., 2015; Vong et al., 2012). Surface engineering further refines delivery: PEGylation reduces non-specific interactions and enhances epithelial distribution in the inflamed colon (Lautenschläger et al., 2013; PLGA, 2026; Tang et al., 2009; Lai et al., 2009); while the theoretical advantage of positively charged NPs for increased mucosal adhesion, particularly in Crohn’s disease, lacks strong experimental validation (Niebel et al., 2012; Han et al., 2012; Gradauer et al., 2013; Manconi et al., 2013). Negatively charged NPs leverage electrostatic interactions with proteins in inflamed tissue, with smaller anionic NPs showing good mucus adhesion (Tirosh et al., 2009; Carlson et al., 1999; Peterson et al., 2002). Redox-mediated targeting utilizes Reactive Oxygen Species (ROS)-responsive nanoparticles that degrade within the inflamed mucosa, releasing payloads and consuming ROS (Chen et al., 2024). Active targeting nanoparticles employs ligands binding receptors overexpressed during inflammation or on specific cells, significantly improving colitis-specific accumulation and enabling strategies (Coco et al., 2013; Xiao et al., 2014; Harel et al., 2011; Xiao et al., 2013).

**FIGURE 5:**
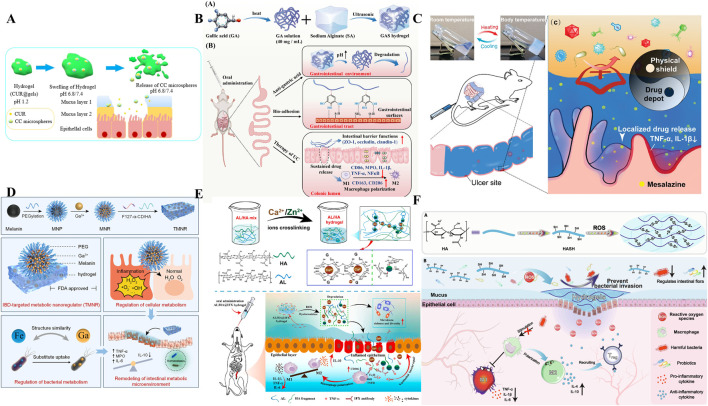
Schematic diagrams of the hydrogels developed for IBD. **(A)** Design mechanism of CUR@gels therapy for IBD disease (Reprinted from Zhang et al. International Journal of Biological Macromolecules, Vol 167, “Carboxymethyl chitosan microspheres loaded hyaluronic acid/gelatin hydrogels for controlled drug delivery and the treatment of inflammatory bowel disease”, Copyright (2021), with permission from Elsevier. DOI:10.1016/j.ijbiomac.2020.11.117) (Zhang et al., 2021) **(B)** Schematic Representation of the Synthetic GAS Hydrogel and the Protective Mechanism on the Colitis Epithelium. (Reprinted from Huang et al. Journal of Agricultural and Food Chemistry, Vol 71, “Mucoadhesive Hydrogel with Anti-gastric Acid and Sustained-Release Functions for Amelioration of DSS-Induced Ulcerative Colitis”, Copyright (2023), with permission from American Chemical Society. DOI:10.1021/acs.jafc.2c07777) (Huang et al., 2023) **(C)** Designofthermo-sensitive hydrogelformulation for UC treatment. (Reprinted from Guo et al. Journal of Materials Chemistry B, Vol 9, “Thermosensitive polymer hydrogel as a physical shield on colonic mucosa for colitis treatment”, Copyright (2021), with permission from Royal Society of Chemistry. DOI:10.1039/d1tb00499a) (Guo et al., 2021) **(D)** Schematic Preparation and Therapeutic Illustrations of TMNR for IBD *via* Regulation of Cellular and Bacterial Metabolism to Restore Intestinal Homeostasis. (Reprinted from Xu et al. ACS Nano, Vol 18, “Metabolic Nanoregulator Remodels Gut Microenvironment for Treatment of Inflammatory Bowel Disease”, Copyright (2024), with permission from American Chemical Society. DOI:10.1021/acsnano.3c11496) (Xu et al., 2024) **(E)** Schematic illustration of the inflammatory repair effects of AL/HA@IFX hydrogel in DSS-colitis mice model. (Reprinted from Huai et al. International Journal of Biological Macromolecules, Vol 249, “Oral colon-targeted responsive alginate/hyaluronic acid-based hydrogel propels the application of infliximab in colitis”, Copyright (2023), with permission from Elsevier. DOI:10.1016/j.ijbiomac.2023.125952) (Huai et al., 2023) **(F)** Schematic diagram of the oral hydrogel precursor solution as artificial mucus barrier for the treatment of inflammatory bowel disease. (Reprinted from Zhang et al. Science Advances, Vol 10, “Artificial mucus layer formed in response to ROS for the oral treatment of inflammatory bowel disease”, Copyright (2024), with permission from AAAS under CC BY license. DOI:10.1126/sciadv.ado8222) (Zhang G. et al., 2024).

Concurrently, stimuli-responsive hydrogels have emerged as highly promising biocompatible carriers for sustained and triggered drug release in IBD. Their hydrophilic networks enable precision release *via* microenvironmental triggers: pH-sensitive hydrogels protect drugs from gastric degradation and release in the higher pH intestine/colon (Zhang et al., 2021; Zhang et al., 2025; Huang et al., 2023). Thermosensitive hydrogels form adhesive barriers or gels at body temperature upon rectal or local administration, enhancing retention (Guo et al., 2021; Xu et al., 2024). and ROS-sensitive hydrogels selectively aggregate at inflamed sites, providing localized drug release and additional benefits like microbial barrier function (Huai et al., 2023; Zhang G. et al., 2024). The schematic diagrams of the hydrogels are listed in Figure 5. Beyond carriers, novel devices like pH-sensitive microneedles demonstrate significant potential for targeted intestinal drug delivery in preclinical models (Trave et al., 2015).

### 5.2 Gastrointestinal tumor

GI cancers represent a major global health burden driven by complex molecular pathogenesis, including genetic alterations, environmental factors, and immunosuppressive microenvironments. Traditional therapies face significant limitations: surgery is often ineffective for advanced or metastatic disease; chemotherapy causes systemic toxicity and poor tumor specificity; while molecularly targeted agents benefit only biomarker-selected subgroups and encounter resistance (Liang et al., 2022). These collective challenges necessitate advanced drug delivery strategies. Targeted delivery systems offer a promising approach to enhance the therapeutic efficacy by increasing intratumoral drug concentration, minimizing off-target toxicity, overcoing resistance mechanisms, and enabling theranostic integration.

Esophageal cancer management employs targeted drug delivery system strategies overcoming key therapeutic hurdles. Light-responsive nanoplatforms provide spatiotemporal control for photothermally triggered therapies including ferroptosis induction and microRNA delivery (Alden et al., 2024; Shi et al., 2025). Biomimetic systems utilizing cancer cell membranes achieve homologous targeting, improving radiosensitization and countering multidrug resistance (Chen et al., 2023). Device-integrated approaches featuring tunable drug-eluting stents and sustained-release polymeric films offer palliative benefits by preventing re-occlusion while reducing systemic exposure (Didamson and Abrahamse, 2021; Fan et al., 2024). Gastric cancer strategies emphasize nanoparticle-based delivery. Active targeting directed at receptors like HER2 significantly enhances tumor accumulation and therapeutic efficacy (Sonzini et al., 2025), biomimetic nanoparticles coated with homologous cancer membranes promote targeted drug delivery and ferroptosis induction (Fan et al., 2024). Advanced approaches include stimuli-responsive nanomotors for deep tumor penetration and synergistic chemo-catalytic-immunotherapy (She et al., 2025), bacteria-mediated platforms exploiting microbial tropism such as *H. pylori*-based systems for targeted delivery and immune activation (Zeng et al., 2024), functional hydrogel systems for controlled release (Zhou et al., 2025; Qian and Xu, 2024) and gastroretentive technologies prolonging gastric residence time to improve bioavailability (Zhou et al., 2025). For colorectal cancer (CRC), Colon-specific or microbiota-mediated delivery is crucial to enhance efficacy and reduce toxicity which exploits colonic bacteria and enzymes for localized release. Microbiota-mediated systems harness colonic bacteria and enzymes for localized drug release and synergistic immunomodulation, demonstrated by spore-conjugated liposomes and inulin hydrogels (Niu et al., 2024; Li et al., 2024).

Pancreatic cancer also necessitates sophisticated delivery to overcome stromal barriers and poor vascularization. Nanoparticle platforms encompass high-capacity mesoporous silica systems (Irinotecan delivery by lipid-coated, 2026), polymeric carriers based on materials like PLGA enhancing drug uptake and cytotoxicity (Crucho and Barros, 2017), and surface-engineered nanoparticles incorporating hyaluronic acid or neutrophil membranes for targeted accumulation and chemoresistance mitigation (Serri et al., 2019; NCBI, 2025). Liposomal systems provide versatile solutions. For instance, anti-tissue factor antibody-conjugated liposomes (TF Ab-Liposomes) dual-target tumor cells and stromal fibroblasts in stroma-rich tumors (Shimizu et al., 2020); a two-step sequential liposomal strategy uses nitric oxide donors (Lip-SNAP) to disrupt desmoplasia followed by gemcitabine-loaded liposomes (Lip-GEM) to enhance penetration and efficacy (Chen et al., 2020); furthermore, a hyaluronic acid-decorated liposome targets CD44^+^ cancer stem cells (CSCs) through ROS-mediated cytotoxicity (Marengo et al., 2019). Micellar formulations, such as Genexol-PM, cremophor-free polymeric micelle paclitaxel, demonstrate clinical promise in combination regimens (Lee et al., 2018; Veeren et al., 2017). Alternative delivery routes include orally administered spore-based systems exploiting the gut-pancreas axis for targeted accumulation (Han et al., 2022).

### 5.3 Peptic ulcer disease (PUD)

Peptic ulcer disease (PUD), a chronic disorder damaging gastric and duodenal mucosa, arises from an imbalance between aggressive factors (gastric acid, pepsin, *H. pylori* infection - present in >90% of duodenal ulcers) and mucosal defense mechanisms. Current therapeutic strategies focus on *H. pylori* eradication using antibiotic combinations, acid suppression (proton pump inhibitors, PPIs), and mucosal cytoprotection. However, conventional oral drug delivery faces significant hurdles: rapid gastric emptying and the ulcer’s microenvironment with low pH and mucus barrier limit local drug retention and concentration at the ulcer site, reducing therapeutic efficacy for healing promotion. Targeted drug delivery systems are thus essential to enhance site-specific drug deposition, prolong mucosal contact time, and overcome these physiological barriers for improved ulcer management (Spósito et al., 2021).

Microparticle systems utilize mucoadhesion and buoyancy for gastric retention. Mucoadhesive microparticle formulations employing polymers like chitosan and alginate adhere to the gastric mucosa, enhancing drug delivery and reducing ulcer severity through anti-inflammatory mechanisms (Hou et al., 2014; Cuña et al., 2001). Complementary floating microparticle systems also achieve prolonged gastroretention, enabling sustained drug release and effective *H. pylori* clearance (Hao et al., 2014). Advancing beyond microscale carriers, Polymeric nanoparticles exploit nanoscale dimensions and surface charge for targeted delivery. Electrostatic interactions facilitate mucosal adhesion and targeted accumulation to ulcerated regions, enhancing ulcer healing compared to free drugs (Abd El Hady et al., 2019; Alai and Lin, 2015). Lipid-based nanoparticles provide advantages for gastroprotective agents. Solid lipid nanoparticles offer enhanced compound protection and controlled release, significantly reducing ulcer area and suppressing inflammatory mediators (Sun et al., 2017). Nanostructured lipid carriers overcome loading and stability limitations of solid lipid nanoparticles, demonstrating superior ulcer reduction and tissue protection (Abdelwahab et al., 2013). Emulsion-based systems enhance solubility, permeability, and gastric protection for both lipophilic and hydrophilic drugs. Water-in-oil microemulsions and organogel-based nanoemulsions accelerate ulcer healing by reducing acid secretion, increasing mucus production, improving bioavailability, and preventing mucosal irritation (Celebi et al., 2002; Lu et al., 2016). Hydrogel systems represent advanced platforms for ulcer management. pH-responsive *in situ* hydrogels provide prolonged gastroprotection *via* sustained drug release and suppression of oxidative stress pathways (Xu et al., 2023). Floating hydrogels significantly extend gastric residence time, enhancing bioavailability and overcoming first-pass metabolism limitations (Murad et al., 2022).

## 6 Summary and outlook

This review has delineated the journey “from bench to bowel” in the field of targeted GI drug delivery, examining both the segment-specific physiological barriers that impede conventional treatments and the fundamental challenges facing advanced systems. Notable advances have been achieved in the design of diverse platforms—such as synthetic and natural nanocarriers, smart hydrogels, and microdevices—utilizing passive, active, and stimuli-responsive targeting strategies. These innovations offer transformative potential for managing refractory GI disorders including IBD, cancers, and peptic ulcers, by enabling site-specific drug release with enhanced local activity and minimized systemic side effects. By integrating barrier characteristics, delivery challenges, and functional strategies into a unified framework, this review provides a translational roadmap for the rational design of GI-targeted therapies. At the same time, the field is moving from proof-of-concept targeting toward a more demanding translational stage in which comparative efficacy, reproducibility, long-term safety, and manufacturability must be evaluated alongside delivery performance.

Beyond general biocompatibility, the core safety risks of oral GI-targeted delivery systems can be grouped into several dimensions. First, at the local level, carriers may disrupt the mucus layer, injure epithelial tight junctions, or delay mucosal healing, thereby aggravating barrier dysfunction rather than relieving it (Wang Y. et al., 2024). Second, some materials or surface chemistries may trigger unintended immune activation, complement responses, or chronic inflammatory signaling, especially under repeated dosing. Third, GI-targeted systems may alter microbial composition or metabolic activity, which is particularly relevant for microbiota-responsive platforms because therapeutic benefit and dysbiosis risk may arise from the same interface (Ren et al., 2023). Finally, translational safety is also influenced by formulation-related variables, including degradation products, residual solvents or cross-linkers, sterilization compatibility (Finbloom et al., 2020), and batch-to-batch reproducibility. Therefore, safety assessment should move beyond single-point cytotoxicity or short-term histology and instead incorporate repeated-dose evaluation, mucosal barrier integrity, immune compatibility, microbiota perturbation, biodistribution, and material fate in a unified framework.

Looking forward, research must focus on designing integrated systems capable of concurrently overcoming multiple GI barriers—such as fluctuating pH, pervasive enzymes, dynamic mucus, and stringent epithelial transport. It is critical to rigorously evaluate the long-term biocompatibility of these materials and their interactions with the gut microbiome, which remains an understudied but vital aspect of safety and efficacy. Three future directions appear especially feasible and important. First, future studies should develop region-aware, sequentially operating delivery systems that are designed around the order of real GI barriers—survival in the upper GI tract, mucus negotiation, epithelial interaction, and finally lesion-restricted release—rather than simply stacking multiple functions in one carrier. Second, dual- and multi-responsive platforms should be benchmarked against simpler single-responsive controls in terms of stability, batch reproducibility, trigger reliability, and therapeutic gain, so that added complexity is justified by measurable translational benefit. Third, preclinical evaluation should move toward safety-by-design and clinically relevant models, including repeated-dose studies, mucus- and microbiota-aware assays, and scalable manufacturing validation, to better predict long-term efficacy and clinical feasibility. Future platforms should incorporate real-time monitoring and feedback mechanisms to achieve adaptive release, paving the way for precision medicine tailored to individual patient profiles. Additionally, scalable manufacturing and rigorous translational models are essential to bridge the gap between laboratory innovation and clinical application. By integrating fundamental principles with engineering insights, this field can accelerate the development of clinically effective, reliable, and personalized GI-targeted therapies.
